# Recurrent Intracranial Ewing Sarcoma

**DOI:** 10.31486/toj.24.0014

**Published:** 2024

**Authors:** Soo Ann Yap, Annabel Helga Sophie Alig, Alena Annbalou Hasenburg, Georg Hilfenhaus, Lars Uwe Stephan, Uwe Pelzer, Sebastian Stintzing, Arndt Stahler

**Affiliations:** ^1^ Department of Hematology, Oncology and Cancer Immunology, Charité Universitätsmedizin, Berlin, Germany; ^2^ Department of Hematology, Oncology and Cancer Immunology, Charité Comprehensive Cancer Center, Charité Universitätsmedizin, Berlin, Germany; ^3^ German Cancer Consortium, German Cancer Research Center (DKFZ), Heidelberg, Germany

**Keywords:** *Chemotherapy–adjuvant*, *neoplasm metastasis*, *radiotherapy*, *sarcoma–Ewing*, *soft tissue neoplasms*

## Abstract

**Background:** Ewing sarcoma is a rare malignant neoplasm that is primarily localized in bone tissues. The prognosis for patients with a newly diagnosed localized Ewing sarcoma has been greatly improved by multimodality treatment. However, treating patients with disseminated or recurrent disease is challenging, with a 5-year overall survival rate of <30%.

**Case Report:** A 17-year-old female with an asymptomatic tumor of the left temple underwent 3 cycles of vincristine, ifosfamide, doxorubicin, and etoposide and achieved partial remission. However, the patient refused further chemotherapy and surgical intervention and was lost to follow-up. After 7 months, the patient presented again with a sizeable tumor on her left temple and worsening symptoms. Chemotherapy with alternating cycles of vincristine, doxorubicin, cyclophosphamide, ifosfamide, and etoposide according to the EURO EWING 2012 trial was initiated. After a positive response, debulking surgery was performed, followed by postsurgical radiation, and partial remission was achieved.

**Conclusion:** Optimal treatment protocols for recurrent Ewing sarcoma are lacking. Treatments are individualized based on the patient's response to treatment and the decisions of tumor boards. Patients with rare tumors such as Ewing sarcoma benefit from multidisciplinary collaboration, resulting in improved quality of care and treatment outcomes.

## INTRODUCTION

Ewing sarcoma is an aggressive, rare, malignant, mesenchymal tumor with high recurrence and mortality rates. Primary tumors occur predominantly in the long bones (47%), pelvis (19%), ribs (12%), and (rarely) skull (4%),^1-3^ but extraosseous manifestation in soft tissues also occurs.^4,5^ Unique disease features include translocation between chromosomes 11 and 22, which results in the formation of the fusion gene EWSR1-FL1.^6,7^ Ewing sarcoma is known to display a heterogeneous clinical presentation; therefore, tumor biopsy is vital to differentiate the disease from other intracranial malignancies.^7^ Treatment strategies include the multidisciplinary approaches of systemic chemotherapy, surgery, and radiotherapy. Nevertheless, an international treatment standard of systemic treatment has not been defined, and the overall survival rate at 5 years for disseminated disease is poor at <30%.^8^ The EURO EWING 2012 (EE2012) trial results published in 2022 reported that the US standard of vincristine, doxorubicin, cyclophosphamide, ifosfamide, and etoposide (VDC/IE) was more effective and less toxic than the European standard of vincristine, ifosfamide, doxorubicin, and etoposide (VIDE) for Ewing sarcoma treatment.^9^

We report the rare case of a patient with a massive extraosseous Ewing sarcoma, intracranial growth, and disseminated metastatic disease who experienced a major clinical response while being treated analogous to the EE2012 protocol and eventually achieved partial remission.

## CASE REPORT

**Figure 1.**
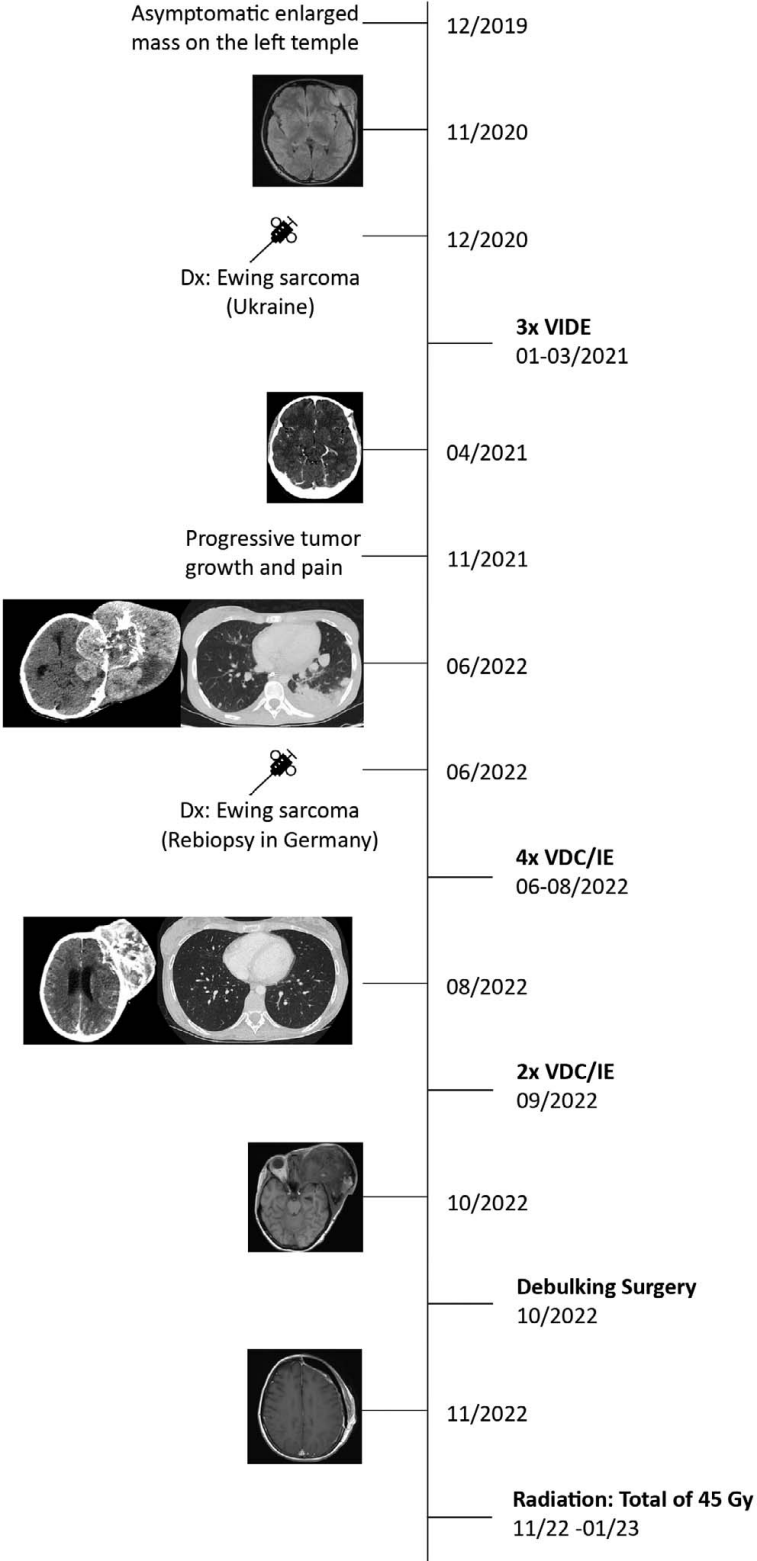
**Timeline of disease progression.** Dx, diagnosis; Gy, gray; VDC/IE, vincristine, doxorubicin, cyclophosphamide, ifosfamide, and etoposide; VIDE, vincristine, ifosfamide, doxorubicin, and etoposide.

Figure 1 is a timeline of the patient's disease course.

**Figure 2.**
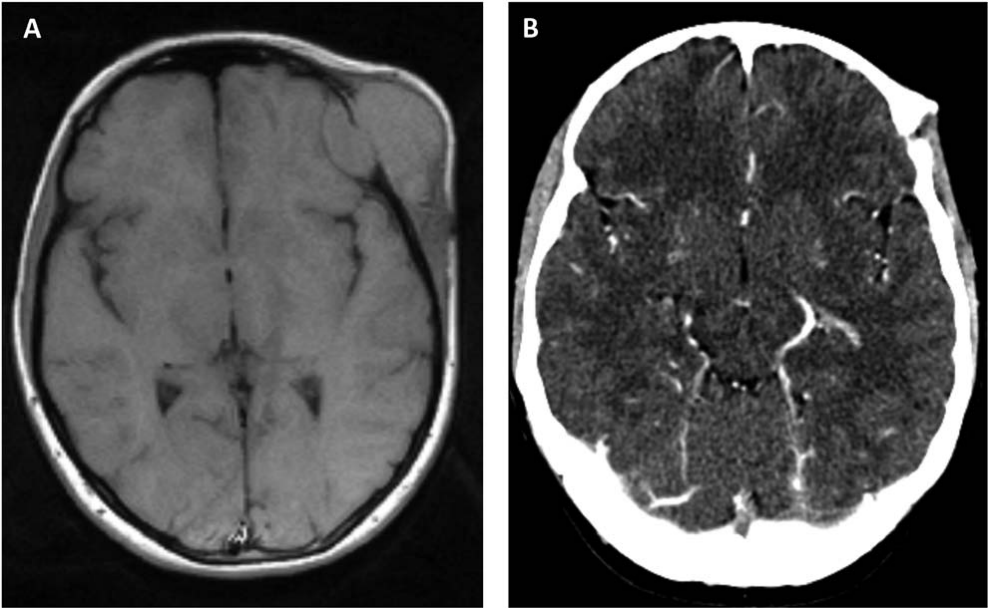
(A) Cranial magnetic resonance imaging shows a large tissue mass (54 mm × 26 mm × 59 mm) in the temporal lobe. (B) Cranial computed tomography shows partial remission (6 mm × 10 mm) after 3 cycles of vincristine, ifosfamide, doxorubicin, and etoposide (VIDE).

In December 2019, a 17-year-old Ukrainian female first noticed an asymptomatic tumor on her left temple. In 2020, computed tomography (CT) and magnetic resonance imaging (MRI) scans showed a tumor measuring 54 mm × 26 mm × 59 mm with infiltration into the skull but without metastatic spread (Figure 2A). After histologic confirmation of Ewing sarcoma, treatment was initiated with 3 cycles of VIDE from January to March 2021. Partial remission was achieved (6 mm × 10 mm, Figure 2B), and surgical resection was planned after the fourth cycle of VIDE. However, the patient refused further treatment and was lost to follow-up.

After continuous disease progression and exacerbated pain, the patient decided to recommence treatment in March 2022. The outbreak of the Russian-Ukrainian war delayed the initiation of treatment until the patient was transported 4 months later to the emergency department at the Charité Universitätsmedizin in Berlin, Germany.

**Figure 3.**
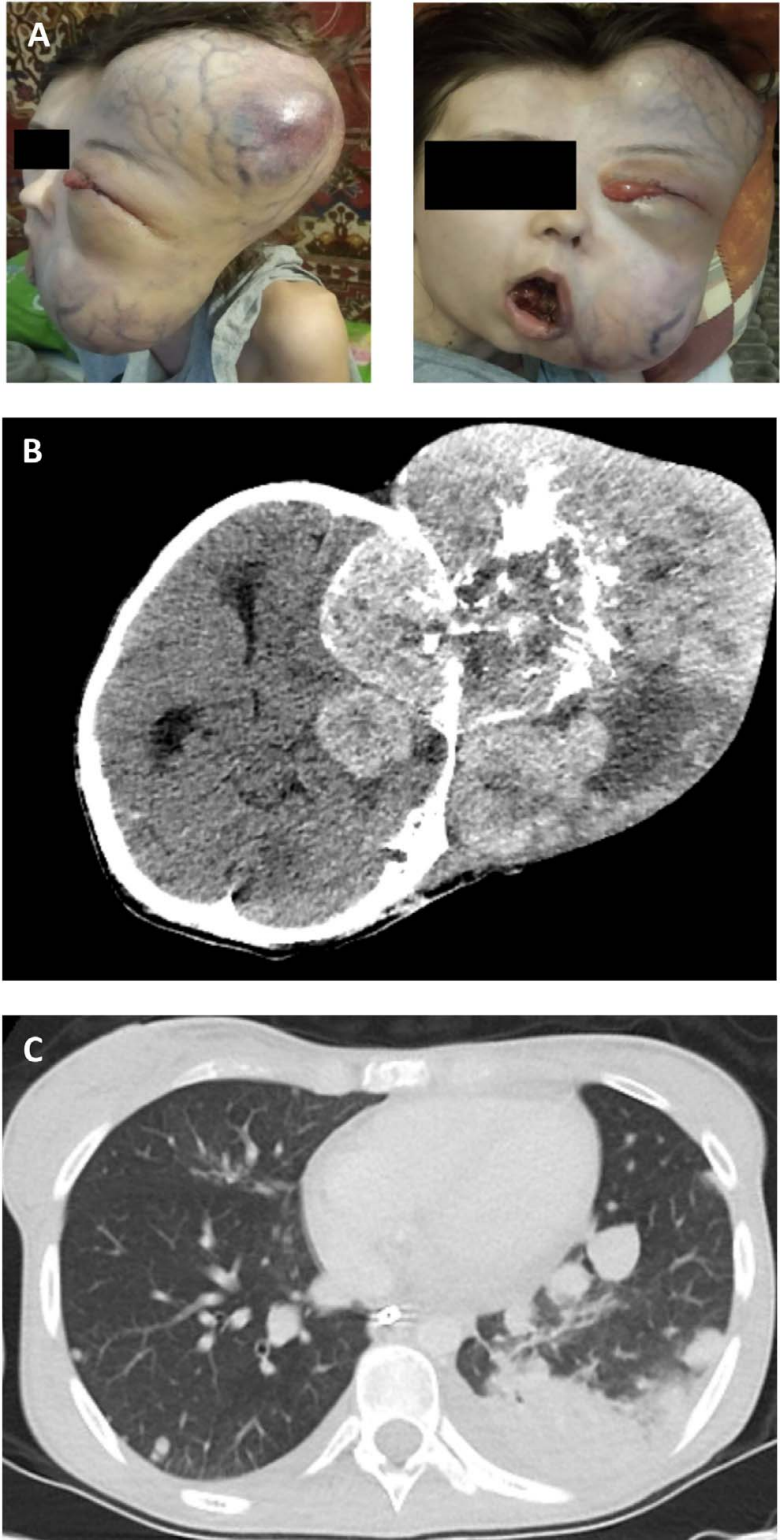
(A) The 20-year-old patient presented with a massive nontender tumor mass on the left temple with visible oral and orbital infiltration. (B) Computed tomography shows a tumor mass of 130 mm × 160 mm × 200 mm in the left hemisphere and (C) pulmonary metastasis.

The now 20-year-old cachectic patient (170 cm, 34 kg, body mass index 11.8 kg/m^3^, Eastern Cooperative Oncology Group Performance Status Scale grade of 4) presented with a massive tumor on her left temple (Figure 3A) that penetrated through the oral cavity but was not associated with neurologic symptoms except for dysphagia. Cranial CT showed a tumor mass of 130 mm × 160 mm × 200 mm with intense vascularization (Figure 3B). Because of the size of the tumor, the pharynx was narrowed, and the left orbital soft tissue was shifted. Surgical resection was not possible.

Dexamethasone (8 mg 3 times daily) was initiated to reduce intracranial pressure, and the dosage was subsequently tapered following improvement of clinical symptoms and intracranial edema shown on follow-up CT. The patient's airway was secured via tracheostomy. Concomitant biopsy histologically confirmed the prior Ewing sarcoma diagnosis. Diagnostic staging procedures (bilateral bone marrow biopsy and CT scan) revealed pulmonary metastases (Figure 3C), pleural carcinomatosis, and suspected peritoneal carcinomatosis.

The multidisciplinary tumor board recommended treatment according to the EE2012 protocol^9^ with restaging after the fourth cycle of chemotherapy with VDC/IE. The recommended treatment was initiated with continuous granulocyte colony-stimulating factor support. Complications of the first cycle were subtle tumor bleeding in the oral cavity, which was treated with tranexamic acid, and generalized epilepsy, which required prophylactic levetiracetam (1,000 mg twice daily, initially intravenously and then administered orally upon regression of the intracranial tumor) and lacosamide (45 mg twice daily, orally).

Headaches, pancytopenia, and recurrent neutropenic sepsis were among the most frequent adverse events. The recurrent neutropenic sepsis resulted from a superinfection of the primary tumor, the tracheostomy wound, aspiration pneumonia, and bilateral pyelonephritis. Consequently, the patient required multiple rounds of antibiotic treatment.

**Figure 4.**
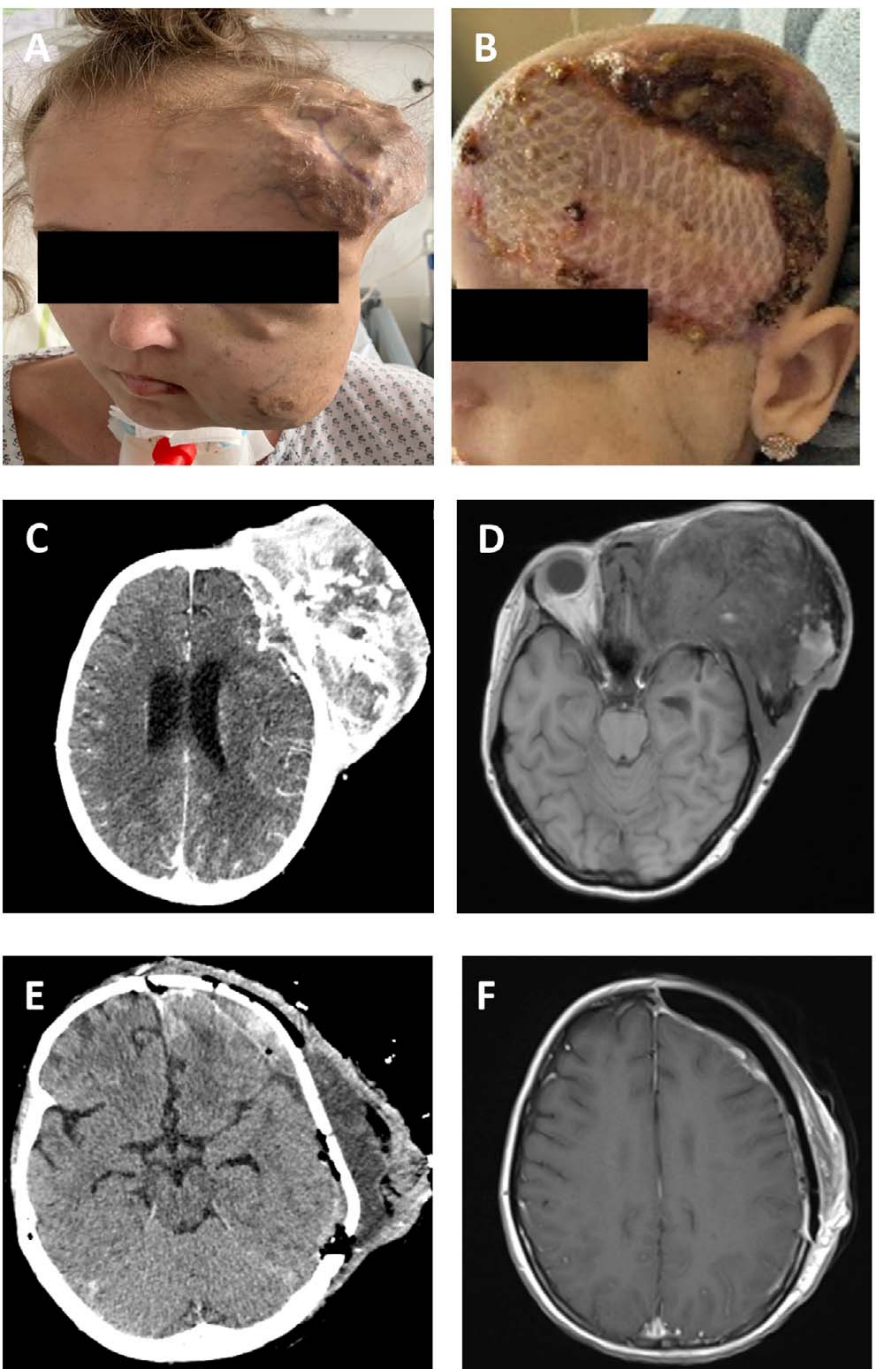
(A) Photograph shows tumor volume reduction and the appearance of necrotic tissue after 4 cycles of chemotherapy. (B) R0 resection was achieved with a successful reconstruction of the calotte. (C) Cranial computed tomography (CT) and (D) cranial magnetic resonance imaging (MRI) show significant shrinkage of the primary tumor (85 mm × 87 mm × 124 mm) after 4 cycles of chemotherapy. (E) Cranial CT shows postsurgical hematoma and fluid retention without evidence of tumor mass. (F) Two months postoperative cranial MRI shows regression of hematoma and fluid retention.

CT scan following the fourth cycle of chemotherapy showed a significant reduction of the primary tumor to approximately 25% of the initial volume (Figures 4A, 4C, and 4D), along with regression of the pulmonary metastasis and pleural carcinomatosis. The previously suspected peritoneal carcinomatosis was no longer detectable. Because of the patient's positive response to chemotherapy, her case was once again presented to the multidisciplinary sarcoma tumor board, and the decision was to follow up with a total of 6 cycles of chemotherapy and evaluation of a secondary resection.

Prehabilitation was implemented to assist the patient who was initially bedridden, impaired by cancer-related fatigue and malnutrition. The prehabilitation regimen included the administration of nutritional supplements, physiotherapy, and psychological support aimed to improve the patient's physical fitness while also enhancing her capacity to withstand stressful events. Oral feeding was challenging because of the invasive tumor in the oral cavity. High caloric nutritional infusion was initially administered to manage the progressive cancer cachexia, followed by regular logopedics, physiotherapy training, and reintroduction of oral feeding upon regression of the tumor in the oral cavity.

Upon completion of the final chemotherapy cycle, 18F-fluorodeoxyglucose positron emission tomography-CT scan demonstrated significant regression of the Ewing sarcoma in the left frontal lobe with lesional calcification and pulmonary metastases.

In October 2022, debulking surgery was performed on the collum mandibulae following hemimaxillectomy; left orbital exenteration; and partial resection of the frontal, parietal, and temporal bone. Microsurgical free flaps were used for the reconstruction of the calotte with a customized left temporomandibular joint and polyether ether ketone implantation (Figure 4B). The postsurgical CT scan (Figure 4E) showed no signs of residual tumor but showed postsurgical hematoma and fluid retention. The hematoma and fluid retention subsequently subsided as seen on the cranial MRI 2 months later (Figure 4F). Pathology confirmed a successful R0 resection and classified the tumor as grade 2 according to the Salzer-Kuntschik classification.^10^

Prophylactic seizure medications were discontinued after successful tumor resection. Adjuvant radiotherapy was conducted with a total dose of 45 Gy. Postradiotherapy staging showed no signs of residual tumor on the cranial MRI; however, lung CT demonstrated progressive pulmonary metastasis, resulting in a partial remission. The patient was hospitalized for a total of 8 months and was discharged after administration of adjuvant radiochemotherapy.

## DISCUSSION

Morphologically, Ewing sarcoma appears as poorly differentiated small round blue cells that can develop in various anatomic locations.^11^ Without treatment, the disease is aggressive as experienced by our patient, in which the initially localized Ewing sarcoma developed into an intrusive large intracranial tumor with distant metastases. Based on the formula V = (13 cm (L) × 16 cm (W) × 20 cm (W))/2, where V is tumor volume, W is tumor width, and L is tumor length,^12^ the cranial tumor volume of our patient was estimated to be 2,080 mL. Tumor volume >200 mL and metastatic disease are associated with a poor prognosis.^13-15^ Despite the poor prognosis in our case, the patient responded well to chemotherapy. After 6 cycles of systemic chemotherapy, the significant reduction of tumor volume permitted surgical reevaluation and subsequent resection.

Systemic chemotherapy is an important part of multimodal treatment of Ewing sarcoma, as initial cytoreduction and postsurgical consolidation are necessary to eradicate microscopic metastatic foci, facilitate sufficient tumor shrinkage, and reduce the likelihood of tumor recurrence.^16,17^ After multiple adjustments in dosage and chemotherapy intervals during the last 30 years, the current EE2012 protocol showed an improvement in 3-year event-free survival with VDC/IE compared to VIDE (67% vs 61%, respectively).^9^ When combined with surgery and irradiation, Krasin et al reported a 5-year local control rate of 89%.^18^ European Society for Medical Oncology guidelines recommend adjuvant radiotherapy with the goal to reduce local recurrence in patients with large tumor volume (>200 mL), inadequate surgical margins, and poor histologic response.^19^

Nevertheless, with a 5-year overall survival of 65% to 75% for patients with localized disease and <30% for patients with distant metastasis, approximately 30% of Ewing sarcoma patients will develop disease recurrence within 2 years.^20,21^

The high recurrence rate and poor disease prognosis upon relapse highlight the urgency of novel therapeutic approaches for Ewing sarcoma. Second-line standard regimens are not yet defined. Current experimental approaches consist of treatment intensification with subsequent hematopoietic stem cell transplantation (autologous or allogeneic) and the evaluation of effective cytotoxic agents.^22-24^ The introduction of comprehensive genomic profiling established the option of broad molecular profiling and targeted treatment based on tumor alterations, particularly for rare cancers.^25^

Ewing sarcoma has a simple genetic arrangement, with 85% of the tumor associated with the t(11;22)(q24;q12) translocation leading to an EWS-FLI1 formation, 5% with t(21;12)(22;12), and the remaining 10% of the cases with a less common translocation inducing a EWS-ERG fusion.^26^ Lurbinectedin, a synthetic tetrahydroisoquinoline alkaloid, has been reported to effectively suppress the activity of oncogenic transcription factor EWS-FLI1.^27^ In a single-arm, open-label, multicenter basket phase II trial, lurbinectedin showed positive efficacy, with 5 of 28 patients achieving disease stabilization for more than 6 months.^28^

A phase II trial of the immunoglobulin G1 monoclonal antibody cixutumumab and mammalian target of rapamycin inhibitor temsirolimus demonstrated a positive response, in which 35% (7/20) of Ewing sarcoma patients responded with stable disease of more than 5 months, and tumor regression was reported in 29% of patients.^29^ Moreover, profiling of 113 patients with Ewing sarcoma revealed secondary ERF and FGFR1 alterations with a prevalence of 7% and 3%, respectively.^30^ Future trials might address these alterations pharmacologically to improve treatment lines and overall survival.

In addition to diagnostics and treatment, comprehensive cancer care requires multidimensional supportive care, taking into consideration the physical, psychological, and social needs of patients.^31^ Nutritional status is an important surgical prognostic marker, as patients with cancer cachexia are associated with poor outcomes after major surgery.^32^ Moreover, malnutrition is associated with the poor outcomes of prolonged hospital length of stay, increased risk of infections, and increased mortality.^33,34^ A meta-analysis of 9 studies involving 914 patients who underwent colorectal surgery showed that the hospital stays of patients who received at least 7 days of nutritional prehabilitation decreased by 2 days.^35^

Participation in a supervised exercise program prior to surgery by patients with rectal cancer who were undergoing neoadjuvant chemoradiotherapy increased the patients’ postsurgical fitness significantly within 3 weeks.^36^ By primary endpoint analysis (ie, 6 weeks postoperatively), patients of the exercise group recovered in terms of fitness and physical activity, while the control group was at high risk of surgical adverse events.^36^ Poor preoperative physical fitness reflects poor physiologic reserves that are associated with postoperative morbidity.^37^ These findings demonstrate the importance of involving multiple disciplines to improve the quality of life, symptom control, and survival of patients with cancer.^38-41^

## CONCLUSION

Management of Ewing sarcoma requires a multidisciplinary effort. Despite available treatment guidelines, the interdisciplinary tumor board should develop a strategy personalized to the patient's condition and tumor growth, especially in cases of relapsed Ewing sarcoma. Along with cancer-specific treatment, patients require comprehensive supportive care to improve symptom burden, quality of life, and outcomes.

## Notes

*This article meets the Accreditation Council for Graduate Medical Education and the American Board of Medical Specialties Maintenance of Certification competencies for Patient Care and Medical Knowledge.*
